# 739 A 14 Year Experience of Pediatric Complex Skin Disorders in a Burn Unit

**DOI:** 10.1093/jbcr/irac012.292

**Published:** 2022-03-23

**Authors:** Heidi M Altamirano, Mark J Johnston, Sam A Miotke

**Affiliations:** Regions Hospital, St. Paul, Minnesota; Regions Hospital, Saint Paul, Minnesota; Regions Hospital, Saint Paul, Minnesota

## Abstract

**Introduction:**

Complex skin disorders including Stevens-Johnson Syndrome (SJS) / Toxic Epidermal Necrolysis (TEN), Ritter's Disease (Staph Scalded Skin Syndrome), and Erythema Multiforme are uncommon, but result in significant injury to pediatric patients. Skin necrosis and desquamation occurs, which in some cases affects mucosa. Gynecologic, ophthalmologic, and dermatological complications also occur. The purpose of this work is to describe epidemiology and management trends in these cases.

**Methods:**

Records were reviewed for all pediatric patients with skin disorders from 2006 - 2019 to evaluate trends in occurrence, age, length of stay, survivability, types of consultants, causative agent, and wound care strategies.

**Results:**

One-hundred percent of pediatric patients were transferred from other hospitals for definitive management by the burn service. The incidence in pediatric patients was 21% compared to 79% in adults. Males were most often affected at 67% compared to 33% in females. The age range was 2-17 years, with an average of 9.2 years. The type most frequently seen was SJS/TEN at 60% of the cases. The total body surface affected ranged from 10-95%. Management of wounds commonly required operative management for dressing changes in children with large body surface area involvement, in addition to ophthalmologic and gynecologic procedures in patients with mucosal involvement. In the subset of patients with SJS / TEN, 100% had ophthalmology consults and 50% were seen by gynecology. The average hospital length of stay was 11.3 days. All children survived.

**Conclusions:**

Complex skin disorders in pediatric patients require a multidisciplinary team approach to care and wound management and benefit from burn service care. Early transfer is beneficial in order to definitively diagnose the specific disorder and prioritize strategies in care such as nutrition, wound care, and psychosocial support.

